# Nano-structure of vitronectin/heparin on cell membrane for stimulating single cell in iPSC-derived embryoid body

**DOI:** 10.1016/j.isci.2021.102297

**Published:** 2021-03-11

**Authors:** Uiyoung Han, Wijin Kim, Hyeonjin Cha, Ju Hyun Park, Jinkee Hong

**Affiliations:** 1 Department of Chemical & Biomolecular Engineering, College of Engineering, Yonsei University, 50 Yonsei-ro, Seodaemun-gu, Seoul 03722, Republic of Korea; 2 Department of Biomedical Science, Kangwon National University, Chuncheon, Gangwon-do 24341, Republic of Korea

**Keywords:** Nanotechnology, Stem Cells Research, Cell Engineering

## Abstract

Individual cell environment stimulating single cell is a suitable strategy for the generation of sophisticated multicellular aggregates with localized biochemical signaling. However, such strategy for induced pluripotent stem cell (iPSC)-derived embryoid bodies (EBs) is limited because the presence of external stimulation can inhibit spontaneous cellular communication, resulting in misdirection in the maturation and differentiation of EBs. In this study, a facile method of engineering the iPSC membrane to stimulate the inner cell of EBs while maintaining cellular activities is reported. We coated the iPSC surface with nanoscale extracellular matrix fabricated by self-assembly between vitronectin and heparin. This nano-coating allowed iPSC to retain its *in vitro* properties including adhesion capability, proliferation, and pluripotency during its aggregation. More importantly, the nano-coating did not induce lineage-specific differentiation but increased E-cadherin expression, resulting in promotion of development of EB. This study provides a foundation for future production of sophisticated patient-specific multicellular aggregates by modification of living cell membranes.

## Highlights

• VTN/HEP nano-coating acts as a flexible individual cellular environment
• VTN/HEP nano-coating stimulates embryoid body to promote its development
• VTN/HEP nano-coating preserves spontaneous cell aggregation

## Graphical abstract

**Figure:**
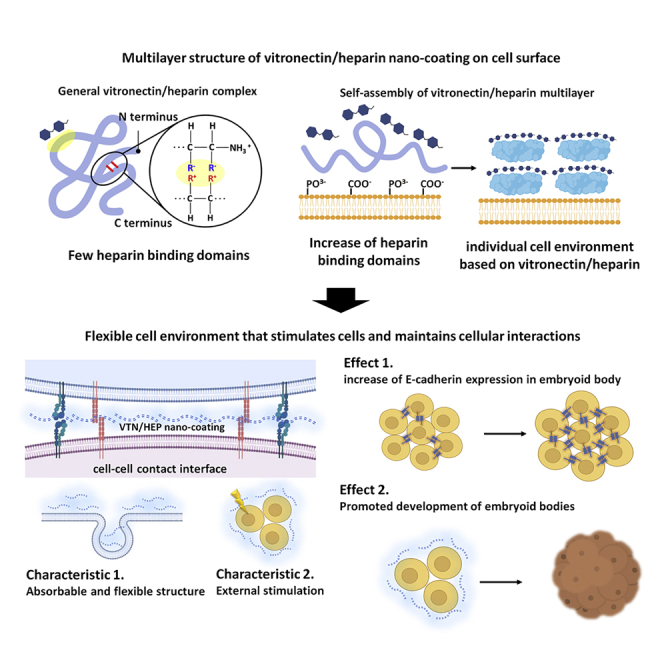

## Introduction

The engineered cell environments that surround single- or multicellular spheroids have a profound effect on their activities and fate. In stem cell research, these tools allow the researcher to design sophisticated extracellular matrices closely mimicking the natural cell characteristics and to enhance the specific cellular properties (Fiorini et al., 2016; Gong et al., 2016; Laranjeira et al., 2017; Minardi et al., 2014; Oda et al., 2005; Sadoshima and Izumo, 1997; Swartz et al., 2001). Thus, numerous studies have elucidated that various mechanical and biochemical stimuli activate specific signal cascades, leading to alterations of the cellular functions (Oda et al., 2005; Sadoshima and Izumo, 1997; Swartz et al., 2001). Based on this previous knowledge, functional hydrogel scaffolds (Fiorini et al., 2016; Laranjeira et al., 2017) and patterned substrates (Gong et al., 2016; Minardi et al., 2014), which enable the regulation of cell differentiation, proliferation, and morphology, have been extensively utilized for various purposes, extending from biological research to biomedical applications. Furthermore, the micro- and nano-structured biomaterials on the cell surface can act as an individual cell environment, inducing the regulation of the cell properties on a single-cell level. Indeed, cell surface engineering including chemical conjugation (Liang et al., 2016), physical adsorption (Leong et al., 2020), and polymeric encapsulation (Mayfield et al., 2014) has been actively studied over the past few years. Among them, the strategy based on the adsorption of biomaterials on the cell membrane has been applied to research aimed at regulating the secretory activity (Lou et al., 2017), survival (Choi et al., 2017a), or differentiation of single cells (Choi et al., 2017b; Han et al., 2020; Hwang et al., 2019). These individual cell environments, denoted as nano-coatings, can be fabricated via chemical reaction or molecular self-assembly of biomaterials, forming various nanostructures on the cytomembrane (Borges and Mano, 2014; Kadowaki et al., 2010). Also, they partially bind to membrane proteins and thus maintain cellular communications, instead of isolating the cells from the surrounding environment (Kozlovskaya et al., 2011; Nishiguchi et al., 2011).

**Figure 1:**
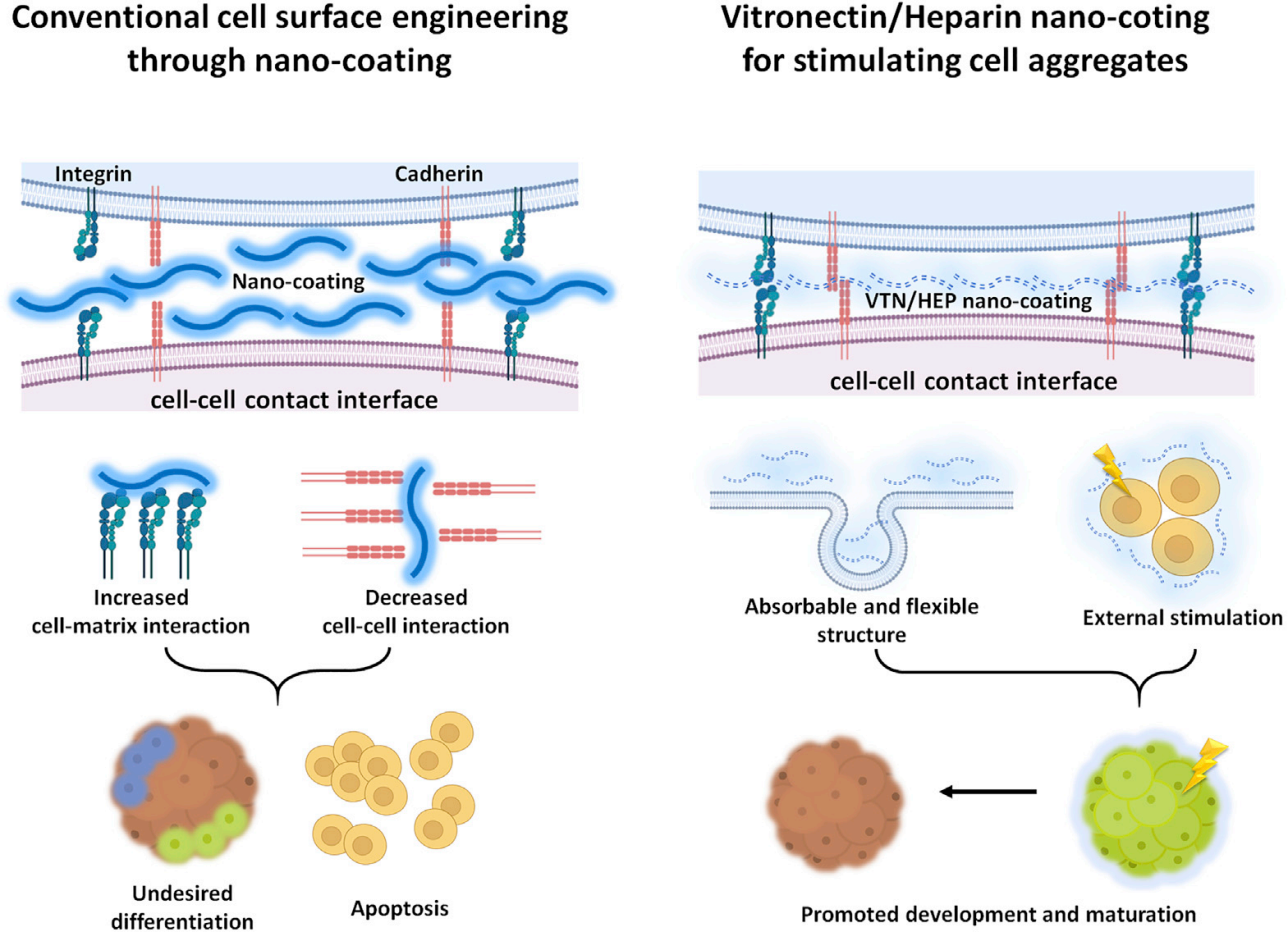
VTN/HEP nano-coating provides a more flexible cell environment compared with conventional nano-coating on cell membrane (A) The schematic illustration represents conventional strategy about cell surface modification through nano-coating. In cell-cell contact interface the presence of nano-coating can increase cell-matrix interaction or decrease cell-cell interaction, resulting in cellular apoptosis or undesired differentiation. (B) The nano-coating fabricated by self-assembly of vitronectin/heparin (VTN/HEP) is an advanced method for controlling single cell in multicellular aggregates without interfering with spontaneous cell activities. In this strategy, the cellular interaction is maintained even in the presence of nano-coating in cell-cell contact interface due to its absorbable and flexible structure. Also, the VTN/HEP nano-coating stimulates single cell and promotes the development and maturation of embryoid body.

However, such strategies are difficult to apply to induced pluripotent stem cell (iPSC), which is a useful tool in advanced biomedical research, from personalized medicine to cell therapies (Nishikawa et al., 2008; Tsuji et al., 2010). For instance, decrease of adhesion signaling during modification of cell membrane can induce apoptosis of the iPSC. Because of the presence of biomaterials adsorbed on the cell membrane, it is also difficult to maintain the balance between cell-matrix and cell-cell interaction in iPSC aggregates and the embryoid body (EB) resulting in undesired differentiation (Figure 1A). As a breakthrough approach for the advancement of iPSC-based research, we tried to confirm the feasibility of the nano-coating method in the regulation of EB properties. To the best of our knowledge, our previous research was the first report that applied the nano-coating method to iPSC culture, in which we established the experimental protocol for maintaining the pluripotency and suppressing the apoptosis of cells during the coating process (Han et al., 2019). However, phenomena in which the expression of the marker proteins was decreased or increased by the nano-coating were simultaneously observed in nano-coated EB. Thus, we thought that it is difficult to control the differentiation of EB with that coating strategy.

In this study, to complement the limitations of our previous research, we suggest nano-coating not with a fibrillar network structure but with a nano-sized globular structure. To be specific, we report a functional nano-coating method promoting the development of EB without the inhibition of spontaneous locomotion of a single iPSC (Figure 1B). For a flexible cellular environment that does not inhibit cell activities, nano-globular coating was fabricated by self-assembly between vitronectin (VTN), a glycoprotein with a relatively low molecular weight, and polysaccharide heparin (HEP). The optimal structure of the nano-coating for promoting the development of EB was established by controlling the amount of VTN/HEP complex adsorbed on cell membrane through layer-by-layer assembly method. As a result, the expression of gene markers related to each of the three germ layers was evenly promoted by the VTN/HEP nano-coating without apoptosis or early differentiation. Our method shows the feasibility of engineering extracellular matrix (ECM) at a single-cell level without interfering with cellular communication. Furthermore, in a multicellular aggregate, if the inner cell can be individually controlled, the establishment, orientation, and maintenance of tissue polarity can be regulated, resulting in the generation of disease- and patient-specific sophisticated tissues.

## Results and discussion

### Structural properties of VTN/HEP nano-coating can be controlled by the number of layers

**Figure 2:**
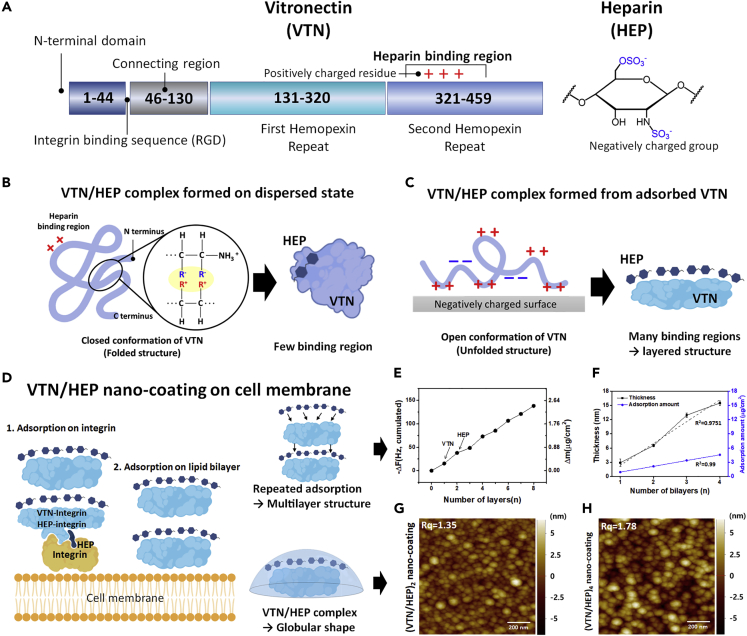
Self-assembled, controllable structure of VTN/HEP nano-coating (A–C) (A) Schematic representation of vitronectin (VTN) and heparin (HEP). The HEP-binding domain is located in the second hemopexin repeat of VTN. Schematic illustration represents the structure of VTN in (B) the dispersed state and (C) the adsorbed state. In the dispersed state, highly acidic residues of VTN in the N terminus interact with positively charged basic residues, resulting in closed conformation. On the other hand, when the VTN is adsorbed on a negatively charged surface, many binding sites are exposed according to appropriate conformation. (D) Scheme of characteristics of VTN/HEP nano-coating on cell membrane. The VTN and HEP are adsorbed on RGD-binding integrin. Also, the positively charged residues of the VTN are adsorbed on the negatively charged phospholipid bilayer. The HEP is adsorbed on the VTN layer through electrostatic interaction and HEP-binding domain in VTN. The structural properties of VTN/HEP nano-coating were analyzed on non-cellular substrate. (E) The result of quartz crystal microbalance (QCM) analysis regarding the adsorption of (VTN/HEP)_4_ nano-coating shows a continuous increase of the accumulative adsorption amount (right y axis), which was calculated from frequency change of the quartz crystal (left y axis) by Sauerbrey equation, with an increase in the number of layers. (F) The thickness (black) and adsorption amount (blue) of VTN/HEP multilayer were plotted versus the number of bilayers. Both graphs show the linear trend line. (G and H) The morphological properties of the surface of the silica substrate coated with the (VTN/HEP)_2_ and (VTN/HEP)_4_ complex was observed using atomic force microscopy. Scale bars = 200 nm. See also Figures S1–S4.

We design the artificial ECM consisting of VTN and HEP, which are components of the ECM, to show the differences with conventional cell scaffold. The integrins, transmembrane proteins on the cell surface, activate intracellular signaling cascades that affect cell survival, proliferation, migration, and matrix attachment (Babic et al., 1999; Giancotti and Ruoslahti, 1999). On the other hand, in the iPSCs culture, the increase of integrin binding induces differentiation and loss of pluripotency (Rowland et al., 2010). In addition, excessive increase in cell-matrix interaction during EB formation causes apoptosis (Kim and Kino-oka, 2014). However, ECM components are still more suitable for coating cell surfaces than other synthetic polymers. Thus, to overcome such limitations of cell surface modification in iPSCs and its aggregates, we fabricated the nano-coating that minimizes integrin-mediated signaling despite the presence of ECM components in the nano-coating. We also thought that effective nano-coating requires the optimization of its structural properties. Thus, we designed the nano-coating with a controllable multilayer structure formed via layer-by-layer self-assembly. In particular, we used VTN and HEP, not fibrillar materials such as collagen, laminin, and fibronectin, which are dominant ECM components, to fabricate a nano-globular structure of the cell surface. Figure 2A shows the structural properties of the building blocks of VTN/HEP nano-coating. The self-assembly of VTN and HEP occurs by electrostatic interaction between the positively charged amino acid in VTN and the negatively charged HEP. However, typical VTN in dispersed state represents a folded structure due to highly acidic residues in the N terminus that interact with the positively charged basic amino acid, resulting in closed conformation, which has one binding residue with HEP (Figure 2B) (Preissner and Müller-Berghaus, 1987). On the other hand, when VTN is adsorbed on a negatively charged surface, many ligand-binding domains are exposed according to appropriate conformation, which allow enough adsorption of HEP. This HEP layer acts as a layer for next adsorption of VTN, and repeated adsorption of VTN and HEP forms a multilayer structure (Figure 2C) (Hou et al., 2017). Thus, a controllable nanoscale artificial ECM consisting VTN and HEP can be formed on the iPSC membrane. In detail, the first VTN layer was formed by its binding with the integrins and the cell membrane itself, wherein a positively charged terminal group of VTN electrostatically interacts with the negatively charged cell surface (Figure 2D). Then, the HEP layer was adsorbed on the VTN layer, which was possible because of the HEP-binding domain including Arg-Gly-Asp (RGD) sequence. Hence, the multilayered VTN/HEP nano-coating was fabricated by repetitive adsorption onto each other. In order to investigate the adsorption properties of the VTN/HEP nano-coating, we repeatedly adsorbed VTN and HEP on the non-cellular substrate. We measured the adsorption amount of each building block using a quartz crystal microbalance (QCM). The results show a linear increase of the adsorption amount until the formation of four bilayers of the VTN/HEP nano-coating, in which a total of 2.48 μg of the VTN/HEP complex was adsorbed on 1 cm^2^ area (Figure 2E). On average, each VTN and HEP layer was adsorbed at a density of 0.21 ± 0.05 μg/cm^2^ and 0.32 ± 0.08 μg/cm^2^, respectively (Figure S1). Regarding the molar ratio, the amount of adsorption was 1:6.74 (VTN:HEP = 62:14 kDa), which was more than the stoichiometric ratio of the VTN/HEP complex (Zhuang et al., 1997), indicating that the HEP-binding site in VTN was increased in an adsorbed state. Thus, Figure 2C indicating the increase in the HEP-binding domain in adsorbed VTN was experimentally proved by the QCM result. Especially, the adsorption amount of VTN and HEP at each coating step did not change much with the increase in the number of layers, indicating that each material adsorbed on the outermost layer provided sufficient binding domain for the adsorption of the next layer. Moreover, the thickness of the VTN/HEP coating increased linearly from 3 to 15 nm with an increase in number of the VTN/HEP bilayers from 1 to 4 (Figure 2F). Thus the VTN/HEP nano-coating on the cell membrane can be controlled by increasing the number of adsorptions of the VTN/HEP bilayers. Then, to investigate the morphological properties of the nano-coating, the surface of the silica substrate, coated with a different number of VTN/HEP layers, was observed using atomic force microscopy. Before coating the VTN/HEP multilayer, the silica substrate was only treated with oxygen plasma to fabricate a negatively charged surface. As a result of analyzing the coated substrate, a uniform nano-globular structure with a diameter of 80–100 nm and a height of 2–4 nm was commonly observed, regardless of the number of layers (Figure S2). The root-mean-square roughness (Rq) of two and four bilayers of VTN/HEP nano-coating was 1.35 and 1.78 nm, respectively (Figures 2G and 2H). In other words, within the range of 2–4 bilayers, the roughness of the nano-coated surface does not change significantly, so it is expected that the structural properties of the cell surface will not be significantly affected. Based on the aforementioned results, we thought that the VTN/HEP nano-coating was successfully formed on a non-cellular substrate and such nano-coating will be formed on the surface of iPSCs through the same driving force.

### VTN/HEP nano-coating acts as absorbable extracellular matrix on the iPSC surface

**Figure 3:**
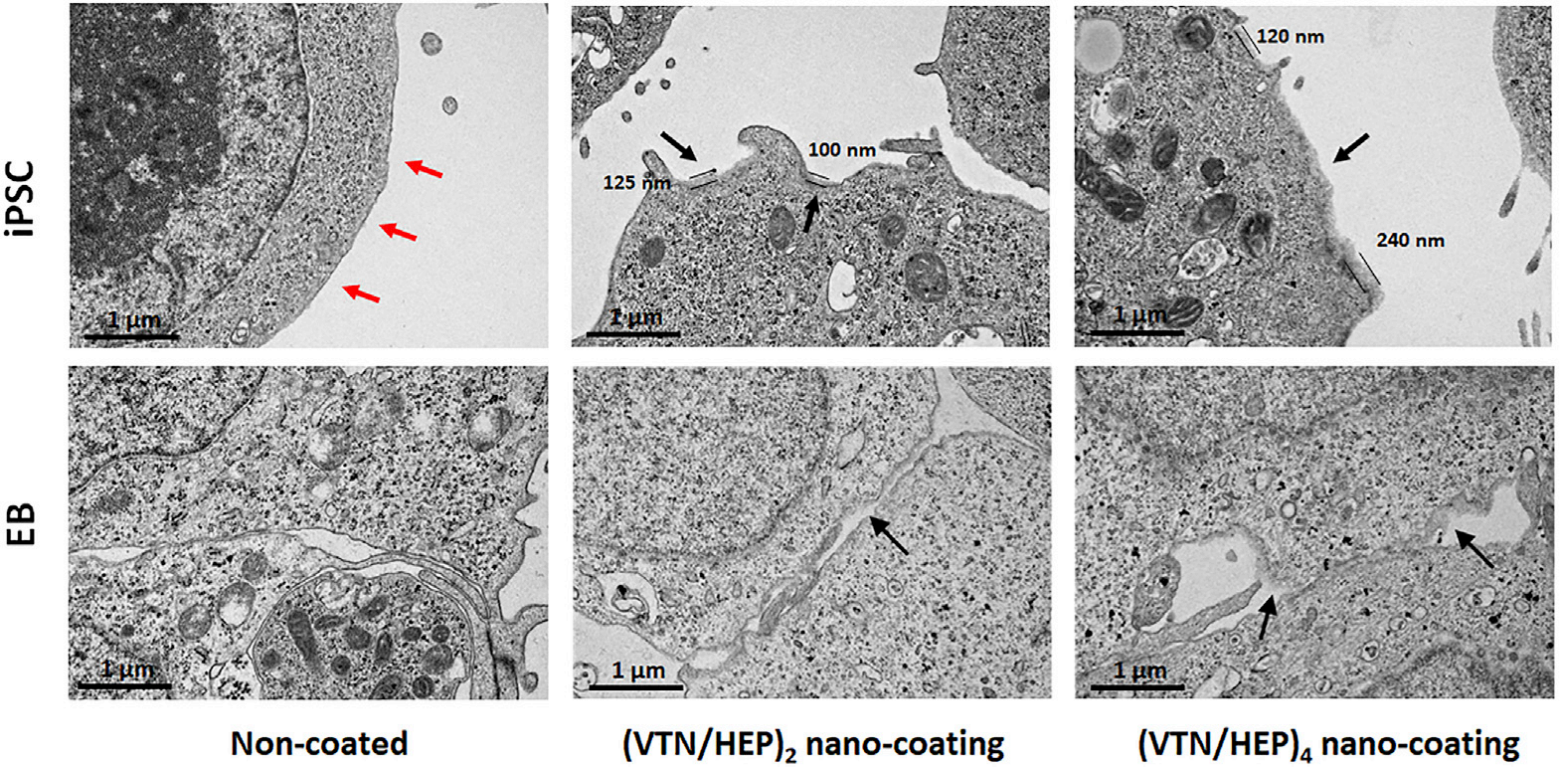
Presence of VTN/HEP nano-coating on iPSC surface and boundary of inner cells Transmission electron microscopic (TEM) images of the non-coated iPSCs and EBs, (VTN/HEP)_2_- and (VTN/HEP)_4_-coated iPSCs, and EBs. The opaque parts (black arrows) on the cell surface represent the presence of the VTN/HEP nano-coating. This is clearly distinguished from the non-coated cell surface indicated by the red arrows. Before taking the TEM images, the iPSCs were fixed immediately after the coating process, and the EBs, formed from the coated-iPSCs, were fixed after 2 days of culture. Scale bars = 1 μm.

To confirm the presence of the VTN/HEP nano-coating on the cell membrane, we observed the construction of the VTN/HEP nano-coating on the iPSC membrane and the boundary of the inner EB using transmission electron microscopy (TEM; Figure 3). Most non-coated cell surfaces were sharply distinguished from the external environment (red arrows). In contrast, the coated cell surfaces showed less clear and thicker layers compared with the non-coated surface (black arrows). The thickness of such layers was 100–125 nm and 120–240 nm, as observed on the iPSC surface coated with the VTN/HEP two-bilayer and four-bilayer complex (denoted by (VTN/HEP)_n_, n = 2, 4), respectively. Although such layers were found in some areas of the inner boundaries of EB, 2 days after coating its coverage was lower than that on the iPSC surface. This difference indicates that the nano-coating is sustainably absorbed into the inner cell during EB formation.

**Figure 4:**
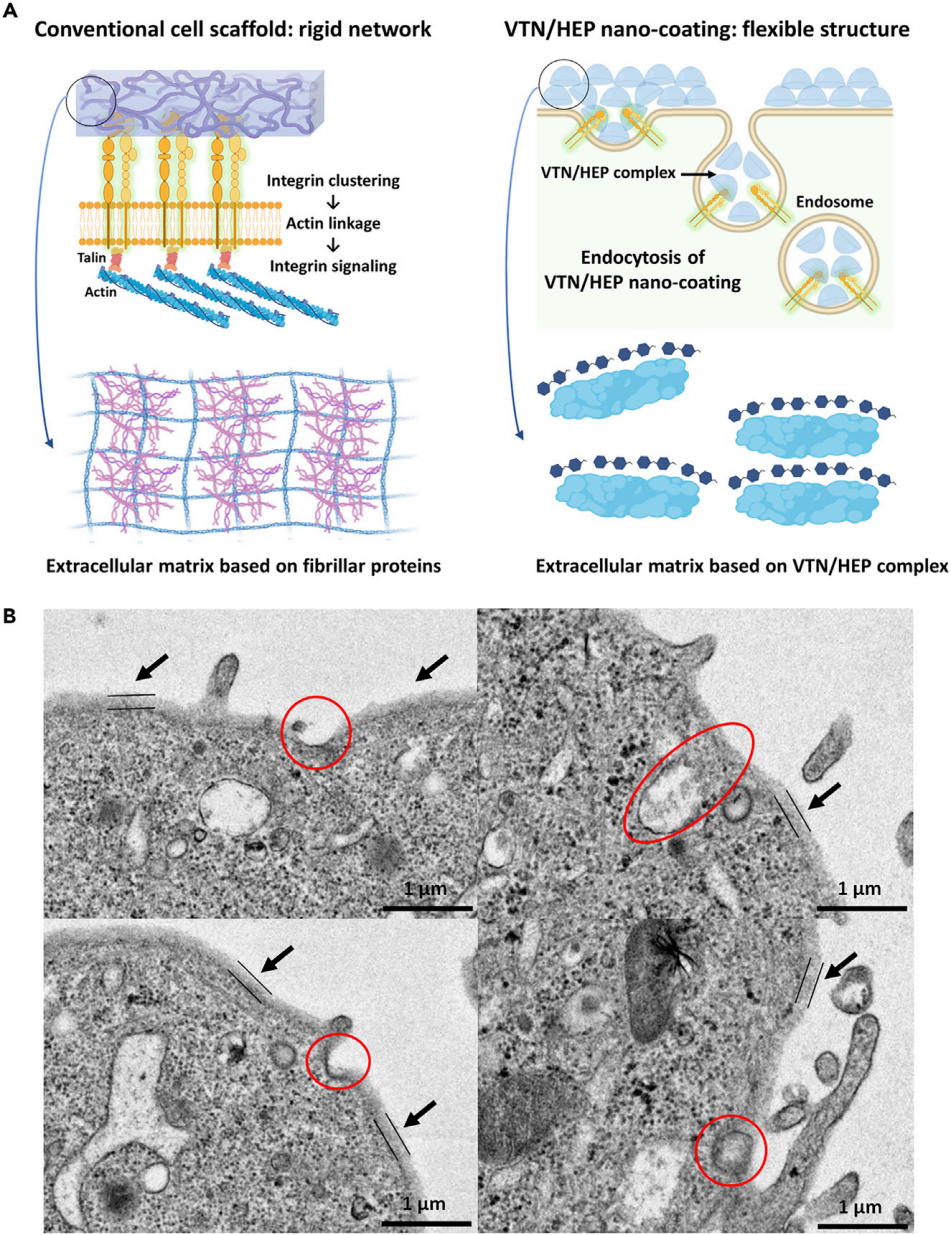
VTN/HEP nano-coating acts as flexible extracellular matrix (A) The schematic illustration on the left shows the characteristics of conventional cell scaffold based on extracellular matrix components. The fibrillar network structure of general extracellular matrix induces an increase of cell-matrix interaction and alteration in cell adhesion properties through integrin clustering. On the other hand, the VTN/HEP nano-coating is composed of aggregates of VTN/HEP complexes with nano-globular morphology. The interaction between each complex is relatively weaker than conventional engineered cell scaffolds. Thus the VTN/HEP nano-coating is partially absorbed into the cell while maintaining the integrin fluidity, rather than inducing alteration of cellular interactions. (B) TEM images of iPSCs coated with the (VTN/HEP)_4_ complex show the absorption of the nano-coating via endocytosis. Several pieces of evidence regarding the endocytosis of the nano-coating, such as the part of the nano-coating with ~190 nm thickness (black arrows), its absorbed state via receptor-mediated endocytosis (red circles), and the endocytic vesicle of the nano-coating, were observed. Scale bars = 1 μm.

The thickness of the VTN/HEP nano-coating on the cell surface differed significantly from that on the non-cellular substrate. We expected that such difference in the thickness of nano-coating was induced by the adsorption of substances other than VTN and HEP. The E8 culture media used for the iPSC coating solution contains various substances such as nutrients and proteins. These materials can be adsorbed together on the cell surface during the assembly of the VTN/HEP coating. As a result of QCM analysis of the VTN/HEP nano-coating, the adsorption amount of the nano-coating in E8 media was higher than that in phosphate buffered saline (PBS), approximately 54% compared with that under phosphate buffered saline (Figure S3). Thus we concluded that the materials in the E8 media are additionally adsorbed onto the iPSC membrane and that they exist in a swollen state, causing a marked difference in thickness. Interestingly, the VTN/HEP nano-coating formed under E8 media conditions remained at 55% of the initial adsorbed amount after 3 h of incubation at 37°C in PBS, whereas the VTN/HEP nano-coating fabricated under PBS conditions remained almost unchanged (Figure S4). Because the adsorption of E8 components and the swollen state of the nano-coating weakens the binding between the layer-by-layer structure, the stability of nano-coating formed from culture media was weaker than that from PBS. Furthermore, an increase in salt concentration due to the E8 media induces the dissociation of VTN-HEP, resulting in a decrease in the binding interactions (Gibson et al., 1999). Nevertheless, we expected that these properties could enhance the advantages of the VTN/HEP nano-coating and hence preserve spontaneous cell activities. We noted that integrins can interact with the VTN/HEP nano-coating. Various integrins that bind to the ECM are the starting point for mechanical stimuli. The higher the density and stiffness of the ECM that can be combined with integrins, the higher the traction stress caused by actomyosin contraction, resulting in various types of signal transduction to cell activities (De Rooij et al., 2005). In other words, the more the integrins form clusters by binding with the elements of the cell environment, the stronger the influence by the external stimuli, resulting in an environment-dependent cell movement. However, we inferred that the nano-globular structure of VTN/HEP on the cell surface does not cause significant alteration in physical and mechanical stimuli. In this case, the integrins bound to the VTN/HEP complex can be in a relatively flexible state, compared with the integrins bound to conventional cell scaffolds, consisting of synthetic polymers, fibrillar materials, and cross-linked complexes (Figure 4A). Hence, we suggest that this phenomenon, i.e., absorption of VTN/HEP nano-coating through receptor-mediated endocytosis, serves as an indirect evidence to support our hypothesis. Notably, between the evenly coated VTN/HEP complex (black arrows), partial areas being absorbed into the cell through receptor-mediated endocytosis were observed (Figure 4B). Furthermore, some endocytic vesicles (~200 nm) encapsulating the VTN/HEP nano-coating were found inside the VTN/HEP-coated iPSCs. If the assembly of VTN/HEP forms a rigid cell environment as fibrillar collagen matrix, it will be absorbed through pinocytosis or phagocytosis (Sprangers and Everts, 2019). In addition, previous studies have revealed that the cellular uptake is significantly reduced when any substance adsorbed onto the cell surface is cross-linked, and its binding interaction becomes stronger (Zhu et al., 2019). From this point of view, we concluded that the VTN/HEP nano-coating, which is partially absorbed into the cell through receptor-mediated endocytosis, can act as a flexible cell environment due to its nano-globular structure and attenuated interaction between VTN and HEP.

### Balance of cellular interactions is maintained even after VTN-HEP nano-coating

**Figure 5:**
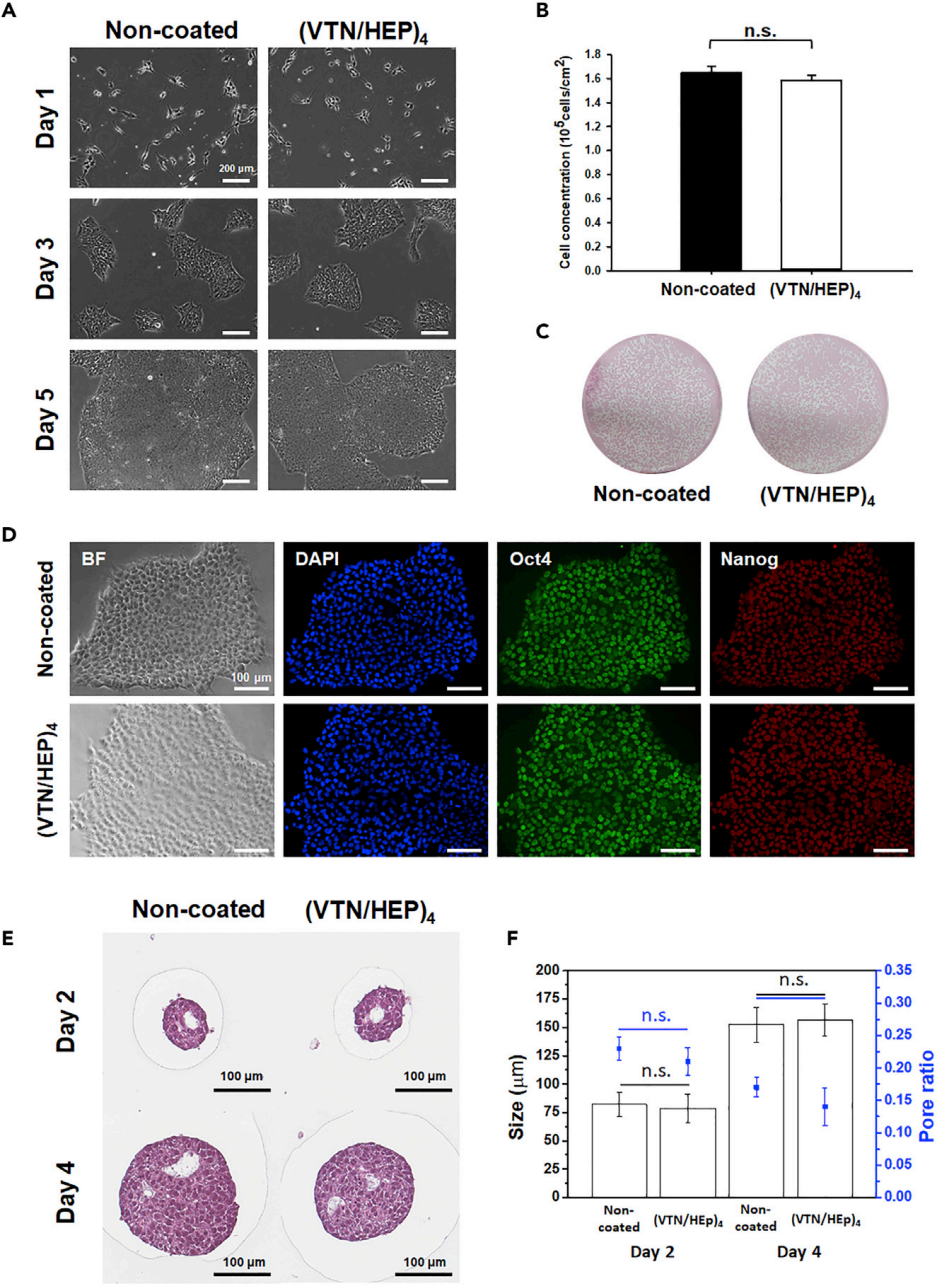
VTN/HEP nano-coating maintains cellular properties affected by adhesion signaling Characterization of the four bilayers of the (VTN/HEP)-nano-coated iPSCs. The non-coated cells also underwent the same coating processes without the addition of the coating biomaterials. (A) There were no significant differences in cell adhesion, colony morphology, and proliferation between the non-coated and (VTN/HEP)_4_-coated iPSCs. Scale bars = 200 μm. (B) On day 5 of culture, cells were dissociated as single cells, and the cell number was determined using a hemocytometer. Statistical significance between non-coated and (VTN/HEP)_4_-coated iPSCs (n.s., not significant) was determined using Student’s t test (n = 3). (C) Comparison of results for alkaline phosphatase staining. Red-colored staining indicates the expression of alkaline phosphatase in the undifferentiated iPSCs. (D) Immunocytochemistry showing the expression of the pluripotency markers, *Oct4* (green) and Nanog (red) in the non-coated and (VTN/HEP)_4_-coated iPSCs. Nuclei were stained with DAPI (blue). Scale bars = 100 μm. (E) Histological analysis for EBs formed from non-coated and (VTN/HEP)_4_-coated iPSCs. After 2 and 4 days of culture for EB formation, the embedded EBs in the agarose block were sliced and the EB sections were stained using H&E. Scale bars = 100 μm. (F) Quantitative analysis for size and pore ratio of EBs. The porosity of the EB section was calculated using ImageJ software. Statistical significance of size and pore ratio between non-coated EB and EB generated from (VTN/HEP)_4_-coated iPSCs (n.s., not significant) was determined using Student's t test (n = 10).

Based on the structural and morphological analysis results of the VTN/HEP nano-coating on the cell surface, we supposed that the mechanical and physical stimulation generated by the VTN/HEP nano-coating would be weak. To prove it, we first investigated the effect of the VTN/HEP nano-coating on iPSC properties. As previously mentioned, the VTN and HEP can bind to RGD-binding integrins (αvβ1, αvβ3, and αvβ5), thereby generating various cellular signals related to adhesion, proliferation, and pluripotency. We compared the proliferation and undifferentiated state of (VTN/HEP)_4_-coated iPSCs (four bilayer structure of the VTN/HEP complex, denoted as (VTN/HEP)_4_) and non-coated iPSCs. The non-coated cells (control group) also underwent the same coating processes as the (VTN/HEP)-coated cells, with no addition of the coating materials (VTN, HEP), to exclude unexpected products resulting from the coating process. As in our previous study (Han et al., 2019), we added a specific inhibitor for Rho-dependent protein kinase (ROCK), Y-27632, in the nano-coating process to block cell apoptosis due to single-cell dissociation. As shown in Figures 5A and 5B, there were no significant differences in cell adhesion, colony morphology, and proliferation between the non-coated and (VTN/HEP)_4_-coated iPSCs. In addition, it was demonstrated that the pluripotent status was stably preserved, even though the iPSCs were coated by the (VTN/HEP)_4_ complex, by investigating the expression of pluripotency markers such as alkaline phosphatase (Figure 5C), *Oct4*, and Nanog (Figure 5D). Then, to confirm that the nano-coating causes physical changes during cell aggregation, we investigated the properties of EBs derived from (VTN/HEP)_4_-coated iPSCs. We induced the (VTN/HEP)_4_-coated iPSCs to form EBs via spontaneous aggregation on an ultra-low attachment plate with a flat bottom. A histological investigation, carried out using H&E staining, revealed that firmly structured three-dimensional EBs were formed, even from the (VTN/HEP)_4_-coated iPSCs, after 2 and 4 days of culture (Figure 5E). Compared with the structure of EBs derived from non-coated iPSCs, the structure of those derived from (VTN/HEP)_4_-coated iPSCs had no significant differences in size and porosity, indicating that coating with the (VTN/HEP)_4_ complex may not lead to any significant changes in the mean distance between neighboring cells (Figure 5F).

Generally, an engineered hydrogel or substrate containing ECM such as collagen, laminin, and VTN induces alterations in the cell-matrix interaction through integrin-ECM binding (Hyysalo et al., 2017; Lou et al., 2018). However, our results indicate that the VTN/HEP nano-coating does not affect such cellular properties. We thought the cellular properties were maintained because the nano-coating was continuously absorbed into the cell. This assumption is based on the fact that the integrin absorbed into cells does not induce cell-matrix signaling and cell adhesion properties. Concretely, when the integrin is activated by binding to the ECM ligand, which is internalized via endocytosis, the ECM is decomposed by lysosomes and an endosomal signal is generated, instead of an integrin-mediated signal (Kechagia et al., 2019; Lobert et al., 2010). Thus, this property of the VTN/HEP nano-coating can be an important advantage in iPSC and EB cultures to maintain the cellular interaction balance.

### VTN-HEP nano-coating induces alterations in E-cadherin expression

Considering the results of cellular properties of the coated iPSCs, we concluded that the VTN/HEP nano-coating adheres to the cell surface without interfering with the binding of the cell membrane proteins or generating effective integrin-mediated stimuli. Then, we speculated that the nano-coating adsorbed onto the cell membrane will temporarily affect the expression of membrane proteins. The membrane proteins such as cadherins and integrins are repeatedly internalized by endocytosis and are recycled back to the cell surface by exocytosis (Le et al., 1999; Maxfield and McGraw, 2004). Moreover, previous results showing that the integrins are stabilized by outside-in activation (López-Ceballos et al., 2016) and that E-cadherin is stabilized by the inhibition of integrin-β1-Src activation (Chen et al., 2016) indicate that external stimuli, integrins, and cadherins are closely related. Therefore, the nano-coating binding with transmembrane proteins has the possibility to induce changes in its expression. Above all, we focused on E-cadherin, a representative member of the cadherin family, which is related to cell-cell interaction and plays a crucial role in EB formation.

**Figure 6:**
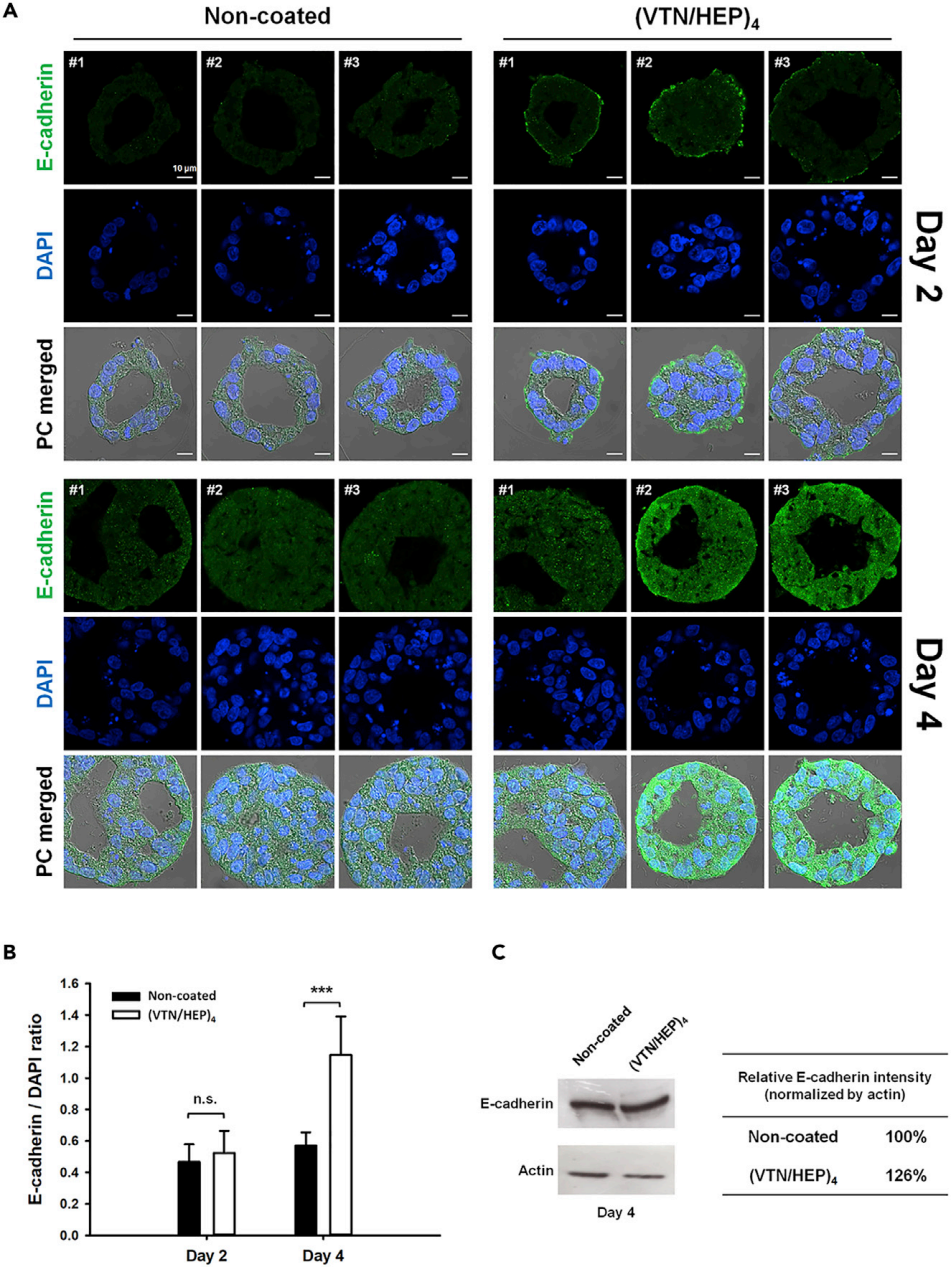
VTN/HEP nano-coating increases expression of E-cadherin The E-cadherin expression in EBs formed from non-coated and (VTN/HEP)_4_-coated iPSCs. (A) Representative images for immunohistochemistry showing the expression of E-cadherin (green) in the EB sections. The nuclei were stained with DAPI (blue). Scale bars = 10 μm. (B) Quantitative analysis of E-cadherin expression in the EB sections. For the area occupied by cells, the fluorescence intensities of E-cadherin and DAPI were determined, respectively, and the E-cadherin/DAPI ratio was calculated using ImageJ software. The statistical significance between EBs formed from non-coated and (VTN/HEP)_4_-coated iPSCs (n.s., not significant and ∗∗∗p < 0.001) was determined using one-way ANOVA and Tukey's method (n = 13). (C) Immunoblot analysis of E-cadherin expression in EBs formed from non-coated and (VTN/HEP)_4_-coated iPSCs. The band intensity for E-cadherin was quantified using ImageJ software and normalized to that of actin. The relative E-cadherin level was expressed as a percentage of the control non-coated iPSCs.

We examined the expression of E-cadherin in the EBs derived from (VTN/HEP)_4_-coated iPSCs and compared it with that of non-coated cells. On day 2 of spontaneous EB formation on the flat-bottomed plates, no significant differences were observed in the overall expression, when E-cadherin was visualized using immunohistochemical (IHC) staining in the sliced EB sections (Figure 6A). However, many of the EBs derived from (VTN/HEP)_4_-coated iPSCs exhibited a high E-cadherin expression at the outermost side, whereas such expression was not found in any of the non-coated cell-derived EBs. On the other hand, the strong E-cadherin expression at the outermost side of EBs in the case of (VTN/HEP)_4_ coating on day 4 of EB formation was not significant, compared with that on day 2. Interestingly, unlike the results of day 2, the overall expression level of E-cadherin on day 4 was higher in the EBs derived from (VTN/HEP)_4_-coated iPSCs than in the EBs derived from non-coated iPSCs. This was also demonstrated via quantitative analysis of the fluorescence intensity of E-cadherin expression on the multiple sliced EB sections (Figure 6B). The immunoblot analysis results also demonstrated that E-cadherin expression was slightly increased by the (VTN/HEP)_4_ nano-coating in the case of spontaneous aggregation, which is coherent with the results of IHC staining (Figure 6C).

We could not yet elucidate the exact reason for the alteration of E-cadherin expression in the nano-coated EB, but it is presumed to be due to the biophysical effect of the nano-coating, such as intercellular interaction and membrane proteins diffusion, not the biochemical stimulation thereof. For proving our assumption, it was confirmed whether the VTN/HEP nano-coating affects the expression of E-cadherin mRNA expression; PCR analysis showed that E-cadherin mRNA expression was not significantly changed in EBs derived from (VTN/HEP)_4_-coated iPSCs (Figure S5). Therefore, we reasoned that the nano-coating increases the temporary distribution of E-cadherin on the surface of cells on day 2. In other words, we suggest that (VTN/HEP)_4_ nano-coating on cell surface may promote intercellular contact among iPSCs in the early stage of spontaneous aggregation, and consequently enhance the stability of the dimeric E-cadherin by limiting the diffusion.

### VTN-HEP nano-coating increases the expression of differentiation markers

Additionally, in order to determine whether the effect of the nano-coating is due to the components of the nano-coating or the physical and structural properties thereof, EBs were formed not only via spontaneous aggregation on the flat-bottomed plates but also via forced aggregation due to gravity on the round-bottomed plates. If cellular properties show the same tendency regardless of the conditions for EB formation, then such results will be due to biochemical factors of VTN/HEP nano-coating. However, if the external physical stimuli (gravity force) are more dominant than the biophysical effect of nano-coating in cell aggregation or intercellular adhesion, the effect of nano-coating will be decreased. Meanwhile, in the previous study, we set the EBs derived from iPSCs immediately after single-cell suspension without the coating process (native) as one control group, and compared them with EBs derived from non-coated iPSCs (Han et al., 2019). As a result of analysis using qPCR, no significant and consistent difference between native and non-coated iPSCs-derived EBs was observed. Therefore, in this study, we compared the EBs derived from non-coated and (VTN/HEP)_4_-coated iPSCs, respectively.

**Figure 7:**
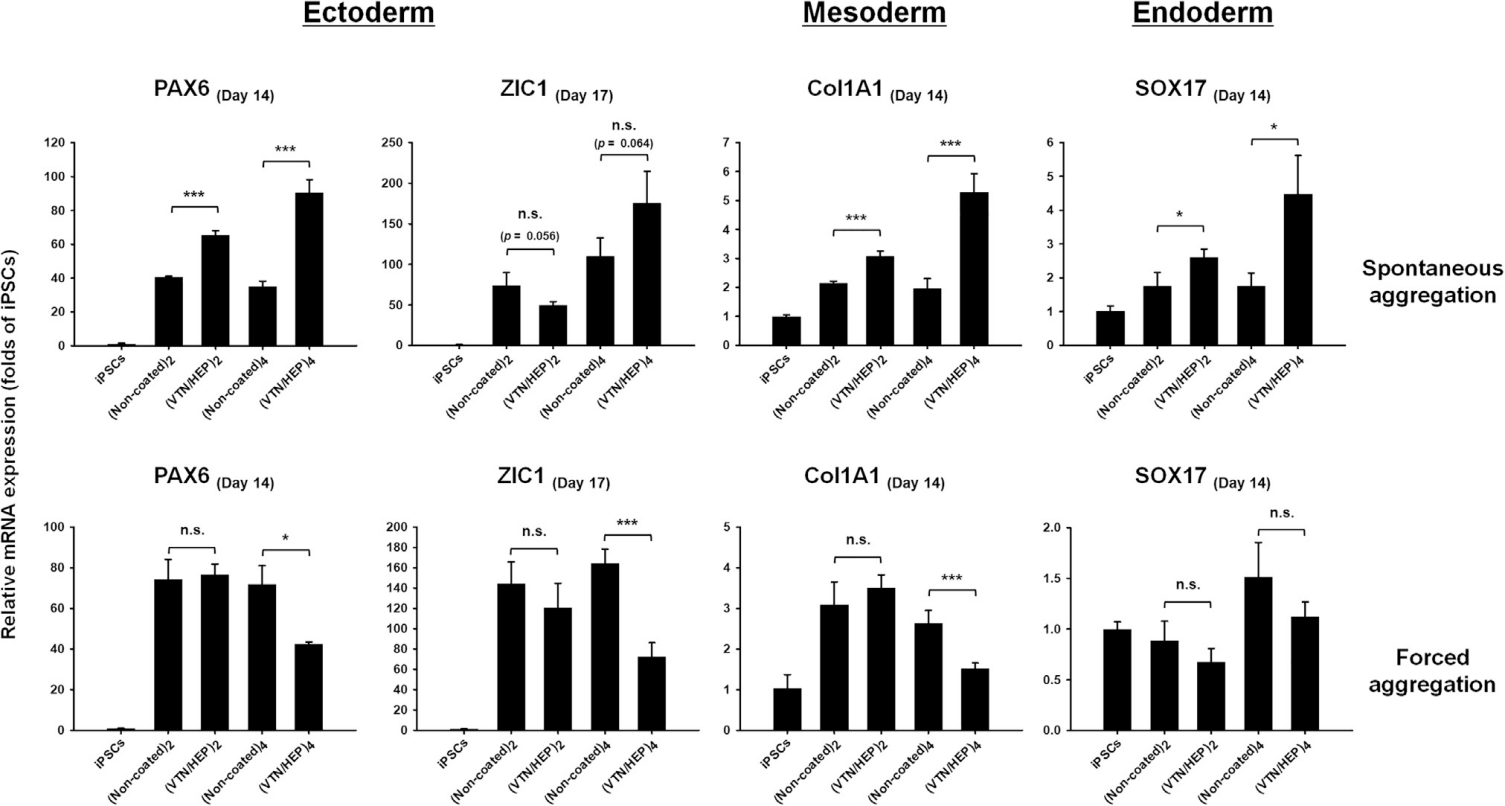
VTN/HEP nano-coating promotes development of embryoid body Quantitative real-time PCR analysis of the differentiation markers for the three germ layers. After formation of EBs via the suspension culture of nano-coated iPSCs in a flat bottom plate (spontaneous aggregation) and in a round bottom plate (forced aggregation) for the first 4 days, the EBs were transferred onto a flat bottom plate and further incubated in suspension culture. Statistical significance of the EB group derived from each number of (VTN/HEP) bilayers-coated iPSCs (n.s., not significant, ∗p < 0.05 and ∗∗∗p < 0.001 compared with the EB group derived from non-coated iPSCs) was determined using Student’s t test (n = 3). See also Figures S5–S7.

We investigated the effect of the nano-coating on subsequent differentiation into the three germ layer lineages. As the suspension culture continued, all iPSCs formed sphere-shaped EBs, and the sizes reached over 500 nm in diameter on day 14. There were no significant differences in the shape, growing rate, and size of the EBs between non-coated and (VTN/HEP)_4_-coated iPSCs in both cases of spontaneous and forced aggregation (Figure S6). However, according to the mRNA expression of lineage-specific markers, which was analyzed using quantitative real-time PCR, (VTN/HEP) multilayer nano-coating has a clear effect on iPSC differentiation through EB formation. Although pluripotency-associated marker gene *Oct4* was downregulated in a similar manner regardless of the nano-coating (Figure S7), the mRNA expression levels of marker genes specific for mesoderm (*Col1A1*), endoderm (*SOX17*), and ectoderm (*PAX6*, *ZIC1*) were higher in the EBs derived from (VTN/HEP) nano-coating-coated iPSCs compared with that in the EBs derived from non-coated iPSCs, in the case of spontaneous aggregation (Figure 7). Interestingly, the increase in the mRNA expression level was more pronounced in the case of coating with four bilayers of (VTN/HEP) nano-coating than with only two bilayers, indicating that the higher the number of repetitions of nano-coating on the cell surface, the more obvious is the effect on EB development. Conversely, when the iPSCs formed EBs via forced aggregation, the mRNA expression levels of marker genes specific for the three germ layers were rather lower in the case of (VTN/HEP)_4_-coated iPSCs than in the case of non-coated iPSCs. These results are presumed to be due to the physical effect of the nano-coating not the biochemical stimulation thereof. Generally, the ECM can stimulate the cell through integrin binding that activates intracellular signaling pathway. Also, it can induce alteration of cell metabolism and differentiation after lysosomal hydrolysis (Gatie and Kelly, 2018). If the VTN and HEP act as biochemical factors, the expression levels of the marker genes in the nano-coated EB should also be increased in forced aggregation conditions. Therefore, the decrease in the effectiveness of the nano-coating under the forced aggregation condition shows that the nano-coating acts as a biophysical factor.

Furthermore, the additional obvious difference between spontaneous and forced aggregation conditions in EB formation is the rate of cellular aggregation. In other words, in spontaneous aggregation conditions, EBs are formed slowly enough to cause a change in E-cadherin expression. In contrast, we inferred that the effect of the nano-coating is relatively reduced because cell-cell adhesion occurs rapidly in forced aggregation conditions. In the case of forced aggregation, the HEP/VTN nano-coating on the cell surface may rather inhibit spontaneous cell-cell interactions. It is evident that these results in the forced aggregation conditions are due to the disturbance of the cell-cell interaction in EB formations via the VTN/HEP nano-coating on the cell surface. The fact that the reduction in the mRNA expression level of marker genes specific for the three germ layers was more significant in the EBs derived from four-bilayer coating than in the EBs derived from two-bilayer coating can support this hypothesis.

In conclusion, we established glycoprotein/polysaccharide-based nano-coating promoting EB development without lineage-specific differentiation by cell-matrix adhesion. The importance of our strategy is that a nano-globular VTN/HEP complex can stimulate development of human iPSC-derived EBs on a single-cell level. The effect of the nano-coating depends on the external physical stimulation, such as gravity force and aggregation rate, and its structural properties such as the number of layers and morphological characteristics. In this study, the four-bilayer nano-coating was the most effective, and its effect was maximized under spontaneous cell aggregation. This strategy is significantly different from conventional functional cell scaffolds fabricated by cross-linking of polymer chains or self-assembly of fibrillar ECM proteins. These properties allow that the inner cell in EB can be stimulated by the nano-coating while maintaining spontaneous cellular communication. Additionally, the E-cadherin expression in nano-coated EBs was increased without a change in mRNA expression, indicating that the VTN/HEP nano-coating has a more prominent effect on the distribution of the plasma membrane proteins than on integrin-mediated signaling or cell-matrix interaction. Meanwhile, although heterogeneous differentiation of the nano-coated EBs was shown in this study, it may also be possible to induce lineage-specific differentiation through various further investigations, such as incorporating cytokines involved in differentiation into a specific lineage within the nano-coating.

The research objectives in tissue engineering are to create functional human tissue equivalents for organ repair and replacement and to develop 3D organoids for disease modeling. To achieve these goals, engineered cellular environments must mimic the physical, chemical, and mechanical properties of *in vivo* tissues and regulate the complex interactions between cells and their microenvironments, which influence the morphogenesis of multicellular aggregates. Especially, spatial and temporal gradients of external stimulation regulate various cellular properties such as proliferation, migration, and differentiation in organoid, multicellular spheroid, and tissue (Miura and Pașca, 2019; Sant et al., 2010). In this context, the control of a single cell in multicellular aggregates can allow the precise designing of a cellular environment to promote development of a specific tissue. So, our approach that stimulates a single cell in multicellular aggregates can be a breakthrough to overcome the limitation in conventional engineered hydrogel scaffolds in biomedical applications and in future clinical trials.

### Limitations of the study

There are limitations and challenges remaining to be addressed in this study. We should investigate a possibility that EB development is promoted by any substance that can be absorbed via receptor-mediated endocytosis on the cell membrane. Also, we expect that the binding between the VTN/HEP nano-coating and specific integrins would also significantly influence the results because ECM-bound integrins can interact with other plasma membrane proteins including cadherin through mechanisms such as crosstalk, antagonism, and synergism (Alghisi et al., 2009; Geiger et al., 2001; Huttenlocher et al., 1998; Huveneers and Danen, 2009). Finally, it is necessary to elucidate the detailed mechanisms of how the structural properties of the nano-coating affect the expression of E-cadherin or specific marker genes of the three germ layers. Further studies about investigation of flexible individual cell environments to control the differentiation of iPSCs and EB in various experimental conditions would be helpful to understand the role of nano-coating on pluripotent stem cell physiologies.

### Resource availability

#### Lead contact

Further information and requests for resources and reagents should be directed to and will be fulfilled by the lead contact, Jinkee Hong (jinkee.hong@yonsei.ac.kr).

#### Materials availability

This work did not generate new unique reagents.

#### Data and code availability

This article includes all analyzed data.

## Methods

All methods can be found in the accompanying transparent methods supplemental file.

## Supplemental information

Document S1. Transparent methods, Figures S1–S7, and Table S1
