# Supplementary material for: Nano-structure of vitronectin/heparin on cell membrane for stimulating single cell in iPSC-derived embryoid body
Source: iScience. 2021 Mar 11;24(4):102297. doi: 10.1016/j.isci.2021.102297 (PMC8022842; doi:10.1016/j.isci.2021.102297)
Supplement: Document S1. Transparent methods, Figures S1–S7, and Table S1 [file mmc1.pdf]

iScience, Volume *24*

Supplemental information

Nano-structure of vitronectin/heparin on cell membrane for stimulating single cell in iPSC-derived embryoid body

Uiyoung Han, Wijin Kim, Hyeonjin Cha, Ju Hyun Park, and Jinkee Hong

Supplemental Figures and Figure Legends

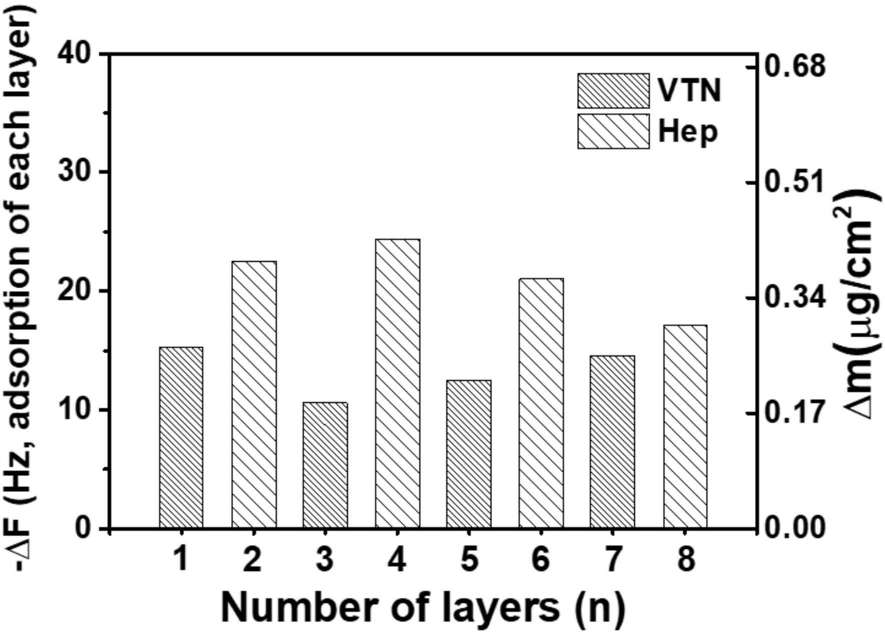

Figure S1. The adsorption amount of each layer in VTN/HEP nano-coating, Related to Figure 2. The result of quartz crystal microbalance (QCM) analysis regarding the adsorption of $(VTN/HEP)_4$ nano-coating, in which a graph representing the adsorption amount of each layer.

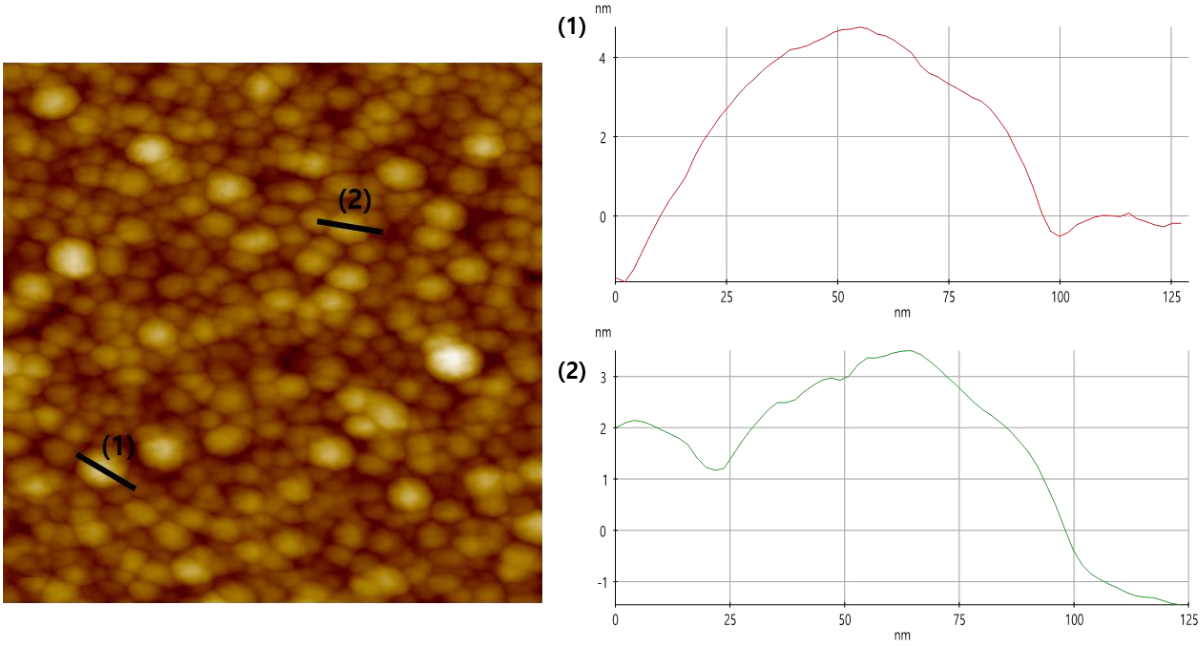

Figure S2. The surface morphology of VTN/HEP nano-coating, related to Figure 2. The nano-globular structure of the VTN/HEP nano-coating was observed using atomic force microscopy (AFM). The size of such structure varies from 20 to 100 nm.

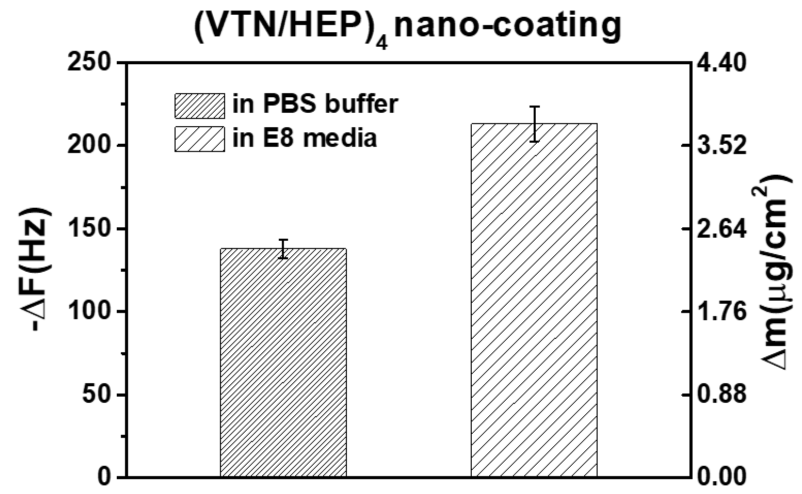

Figure S3. Total adsorption amount of VTN/HEP nano-coating with different solvent condition, Related to Figure 2. Total adsorption amount of $(VTN/HEP)_4$ nano-coating was measured using a quartz crystal microbalance. To confirm the change in adsorption properties by the solvents, the VTN/HEP nano-coating was formed in PBS buffer or E8 media solvent conditions, respectively.

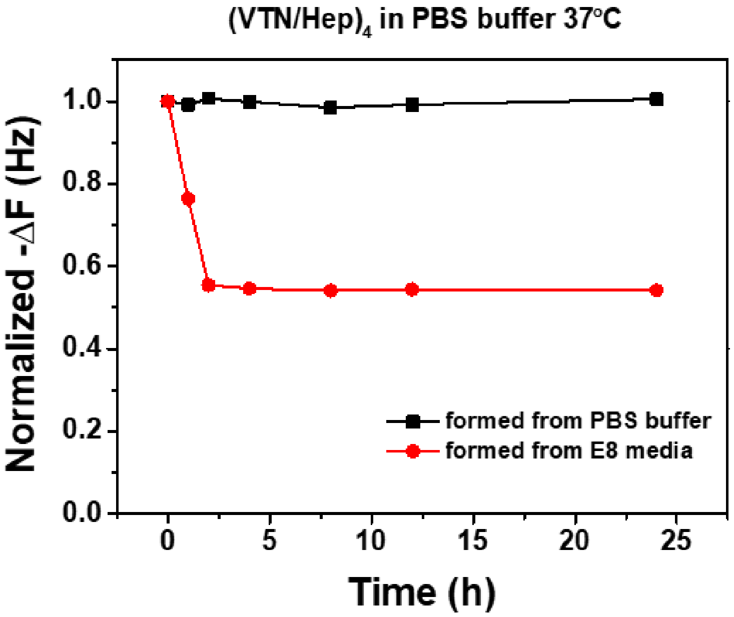

Figure S4. The structural stability of VTN/HEP nano-coating, Related to Figure 2. Stability of VTN/HEP nano-coating was estimated by the change of frequency of the QCM electrode coated with the $(VTN/HEP)_4$ complex during PBS buffer or E8 media incubation at 37℃.

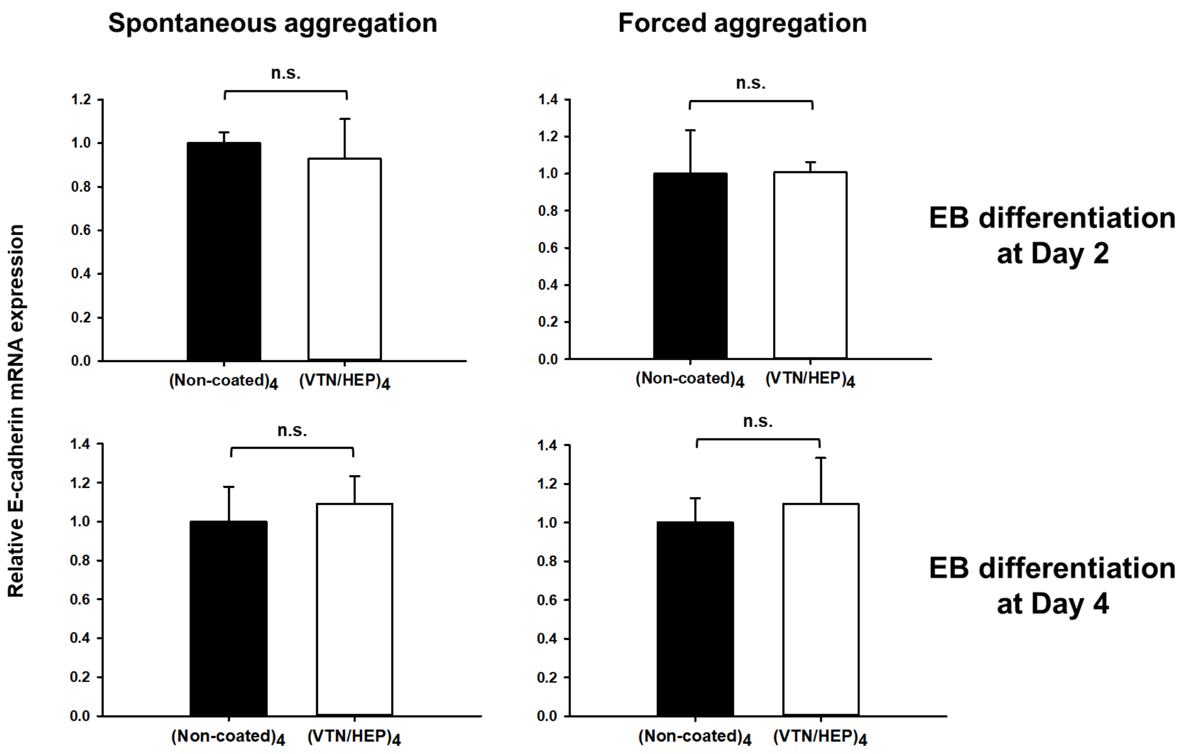

Figure S5. Quantitative real-time PCR analysis of E-cadherin, a cell-cell interaction marker, Related to Figure 7. After formation of EBs using a suspension culture of non-coated and $(VTN/HEP)_4$-coated iPSCs in a flat bottom plate (spontaneous aggregation) and in a round bottom plate (forced aggregation) for 2 and 4 days, respectively, the total RNA was prepared and the mRNA expression level of E-cadherin was measured. The statistical significance of the EB group derived from $(VTN/HEP)_4$-coated iPSCs (n.s., not significant compared to the EB group derived from non-coated iPSCs) was determined using Student's *t*-test (n = 3).

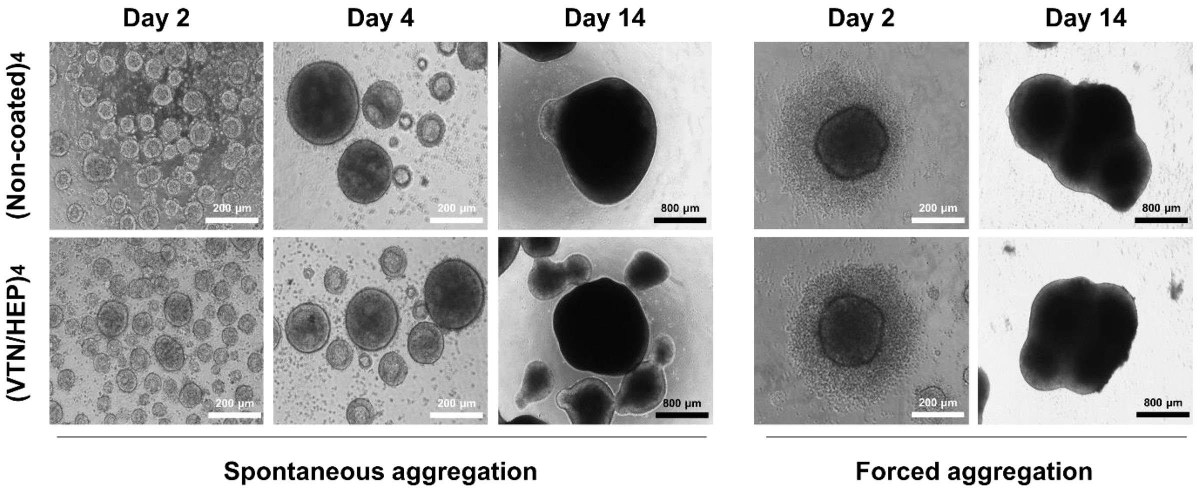

Figure S6. Representative images of EBs formed from the four bilayers of VTN/HEP-coated iPSCs, Related to Figure 7. After spontaneous aggregation and forced aggregation for the first 4 days, the EBs derived from non-coated and $(VTN/HEP)_4$-coated iPSCs were transferred into a flat bottom plate and further incubated in a suspension culture. The date above each image column indicates the total culture period of EB formation.

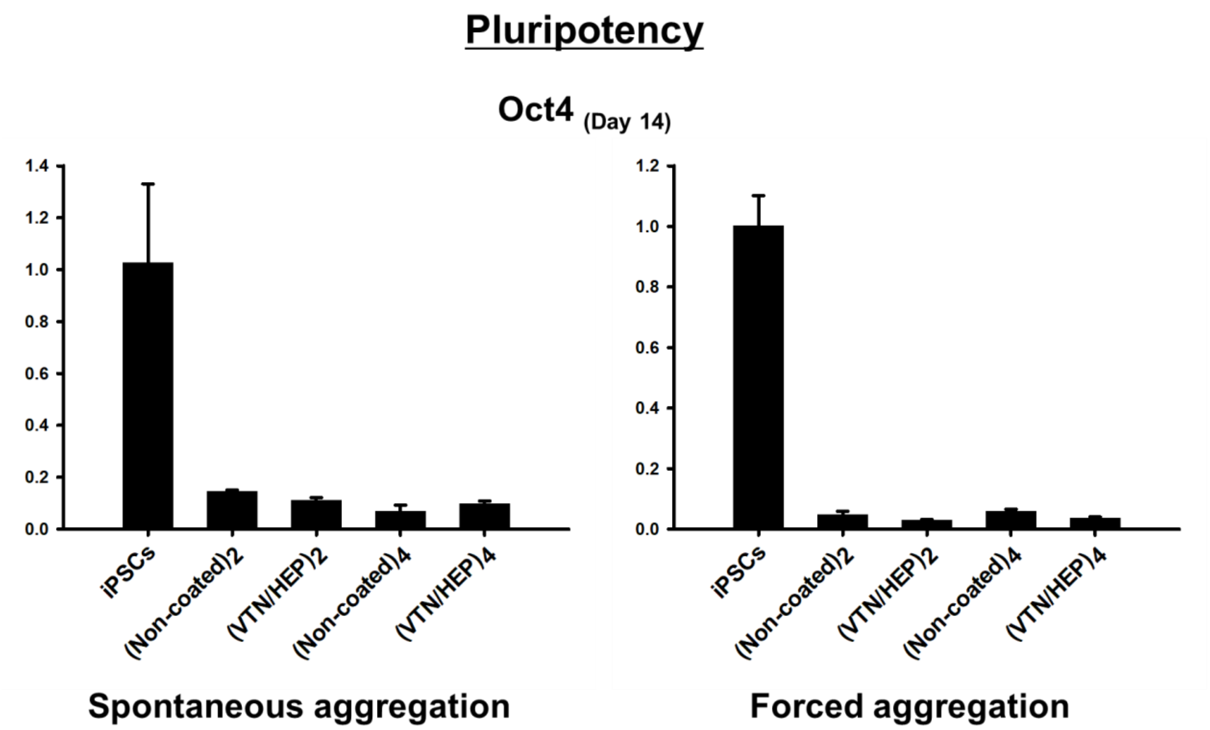

Figure S7. Quantitative real-time PCR analysis of pluripotency marker *Oct4*, Related to Figure 7. After spontaneous aggregation and forced aggregation for the first 4 days, the EBs derived from non-coated and $(VTN/HEP)_4$-coated iPSCs were transferred into a flat bottom plate and incubated in a suspension culture for further 10 days.

Table S1. List of primers used for quantitative real-time PCR analysis, Related to Figure 5,6,7

Gene	Primer	Sequence (5′-3′)
GAPDH	Sense	GTC AGT GGT GGA CCT GAC CT
	Antisense	TGC TGT AGC CAA ATT CGT TG
PAX6	Sense	GTC CAT CTT TGC TTG GGA AA
	Antisense	TAG CCA GGT TGC GAA GAA CT
ZIC1	Sense	AGC GAC AAG CCC TAT CTT TG
	Antisense	CGT GGA CCT TCA TGT GTT TG
Col1A1	Sense	GGA CAC AAT GGA TTG CAA GG
	Antisense	TAA CCA CTG CTC CAC TCT GG
SOX17	Sense	CAT GAC TCC GGT GTG AAT CTC
	Antisense	CAG TAA TAT ACC GCG GAG CTG
Oct4	Sense	GAA GGA TGT GGT CCG AGT GT
	Antisense	GTG AAG TGA GGG CTC CCA TA

Transparent Methods

Fabrication of the nano-coating on non-cellular substrates

Before modification of living cell membrane with nano-coating, it was fabricated on solid flat substrate with negatively charged surface. The surface of silicon wafer and quartz crystal were modified by O_2 plasma (CUTE-1B, Femtoscience, Yongin, Korea) treatment for 2 min leading to the formation of hydrophilic functional groups. The multilayer structure of nano-coating was formed by layer-by-layer dip-coating method. Firstly, a VTN layer was formed by immersing the substrate in VTN coating solution (0.2 mg mL^{-1}) for 10 min. Then, the substrate was rinsed three times (2, 1, and 1min) by PBS buffer solution without drying. While keeping the substrate moist, a HEP layer was adsorbed onto the substrate in the same way (1 mg mL^{-1} of HEP coating solution). The thickness of nano-coating on substrate was measured using a contact profilometer (Dektak 150, Veeco, Oysterbay, NY, USA). The adsorption amount of each layer in nano-coating was measured using quartz crystal microbalance (QCM; QCM200, Stanford Research Systems, Sunnyvale, CA, USA). The Sauerbrey equation was used for conversion of oscillation frequency of crystal to mass. The surface morphology of nano-coating was observed using an atomic force microscopy (AFM; NX-10, Park Systems, Suwon, Korea).

Fabrication of the nano-coating on induced pluripotent stem cells

All solutions for the nano-coating on iPSC membrane were sterilized using 0.2-μm syringe filters. The solutions were prepared by dissolving VTN (0.2 mg mL^{-1}) or HEP (1 mg mL^{-1}) in E8 medium with 10 μm ROCK inhibitor, respectively. Harvested 2×10^6 iPSCs were suspended in the 1 mL VTN solution with mild pipetting. The concentration of cells does not exceed 1×10^8 cells mL^{-1}. The Then the VTN solution was removed by centrifugation at 120 × g for 3 min and iPSCs were washed twice with E8 medium. Such process was repeated with the HEP solution, resulting 1 bilayer nano-coating on iPSC surface. The number of bilayer in the VTN/HEP nano-coating is the number of the times in repeated process. The process was repeated until the desired number of the times this process nano-coating layers had been fabricated.

Transmission electron microscopy imaging

After fabrication of the nano-coating on iPSC surface, such cells were immersed in Karnovsky's fixative (2% glutaraldehyde, 3% paraformaldehyde in 0.2 м cacodylate buffer) 2 h at 4°C. After washing with 0.05 м sodium cacodylate, the cells resuspended in post-fixation solution (1% OsO_4) for 2 h at 4°C. Then, the collected cells were rinsed with deionized water and were stained with 0.5% uranyl acetate. After dehydration, the cells were embedded in epoxy resin in drying oven at 70°C for 24 h. The thin sections were obtained using ultramicrotome (EM UC7, Leica Camera, Wetzlar, Germany), which were then mounted on copper grids. Final samples on the grids were observed using a transmission electron microscope (TEM; JEM1010, JEOL, Tokyo, Japan).

Human induced pluripotent stem cell culture and embryoid body formation

As described in our previous studies, a human iPSC line, generated by introducing four transcription factors (Oct4, Sox2, c-Myc, and Klf4) into human dermal fibroblasts using Sendai virus, was obtained from the National Stem Cell Bank of Korea (Korea National Institute of Health) for use in the present study. The iPSCs were cultured in TeSR™-E8™ medium (StemCell Technologies) with daily medium replacements. At 80%–90% confluency, the cells were detached using 0.5 mm EDTA and plated onto a Matrigel™ (Corning, Corning, NY, USA)-coated culture plate. For EB formation, the iPSCs were first dissociated as single cells by gently pipetting after EDTA treatment. In the case of spontaneous aggregation, the cells were plated onto Ultra-low attachment 24-well plates (flat bottom, Corning) at a density of 3×10^4 cells cm^{-2} and further cultured in EB medium (AggreWell™ EB formation medium,

StemCell Technologies) for 4 days. Conversely, in the case of forced aggregation, the cells were plated onto Ultra-low attachment 96-well plates (round bottom, Corning) at a density of 1×10^4 cells cm^{-2} and cultured for same period. In contrast to the flat-bottomed plate, the iPSCs were gathered in the center of the round-bottomed plate by gravity and start to form EB clumps. Subsequently, the EB clumps were transferred onto Ultra-low attachment 24-well plates (flat bottom, Corning). During EB formation, the medium was exchanged every 2 days in all cases.

Histology

To visually assess the EBs, they were harvested at days 2 and 4 of culture and stained with hematoxylin and eosin (H&E). Before fabricating the cell blocks, the EBs were fixed in a 35% formaldehyde solution and incubated for 12 h at 20-25°C. To embed the EBs in agarose, 1% agarose solution and the EBs were mixed evenly.

The EB slides for H&E staining were prepared using the conventional protocol.(Carpenedo et al., 2007) The EB blocks were dehydrated using a series of graduated alcohol solutions (70%–100%) and xylene and were embedded in paraffin. Then, 5-μm paraffin sections were cut using a rotary microtome. The sections were deparaffinized, and antigen retrieval was conducted by incubating the slides with 1.5 n HCl at 37°C for 15 min, followed by two 5-min rinses using 0.1 m borax buffer (pH 8.5). The sections were stained with H&E, and the size and pore ratio of the EBs were calculated using Image J software (National Institute of Health, Bethesda, MD, USA).

Immunostaining analysis

For immunocytochemistry, the cells were fixed with 4% paraformaldehyde (PFA) for 15 min, and subsequently permeabilized with 0.25% Triton X-100 in phosphate-buffered saline (PBS). After blocking with 3% bovine serum albumin (BSA)-containing PBS-T (0.1% Tween-20 in PBS), the cells were incubated with the anti-Oct4 and anti-Nanog polyclonal antibodies (Santa Cruz Biotechnology, Santa Cruz, CA, USA) at 4°C overnight, and subsequently, with Alexa Fluor 488- and 594-conjugated secondary antibody (Thermo Fisher Scientific, Waltham, MA, USA) at 20°C for 1 h. Following incubation with 4′,6-diamidino-2-phenylindole (DAPI; Sigma-Aldrich, St. Louis, MI, USA), for nuclei staining, cell images were obtained using a fluorescence microscope (Leica Microsystems, Wetzlar, Germany). For histological analysis, the paraffin was first removed by repeatedly washing with xylene. After rehydration in graded ethanol solutions (100%–0%), the sliced EB sections were fixed with 4% PFA for 15 min, followed by permeabilization with 0.25% Triton X-100 in PBS. After blocking with 3% BSA-containing PBS-T, the sections were immunostained using anti-E-cadherin polyclonal antibody (Santa Cruz Biotechnology) and Alexa Fluor 488-conjugated secondary antibody (Thermo Fisher Scientific). Following incubation with DAPI, the fluorescence was observed using confocal laser scanning microscopy (Carl Zeiss, Oberkochen, Germany).

Alkaline phosphatase staining and immunoblot analysis

Alkaline phosphatase (ALP) staining was perfomred according to the manufacturer’s instructions (Stemgent, Cambridge, MA, USA). Briefly, an ALP staining solution was added to cover each well plate after the iPSCs were fixed with 4% PFA for 15 min, and the cells were incubated at 20°C for another 15 min. Following the aspiration of the remaining solution and washes with PBS-T, PBS was added to avoid drying and the ALP-stained cells were observed. For immunoblot analysis, EB-forming cells were lysed using Radioimmunoprecipitation assay (RIPA) buffer (50 mm Tris-HCl, pH 7.4, 150 mM NaCl, 1% Triton X-100, 0.1% sodium dodecyl sulfate (SDS), and protease inhibitor cocktail) at 4°C for 1 h. After centrifugation at 12,000 rpm, the proteins in the soluble cell lysate were resolved using SDS-polyacrylamide gel electrophoresis (SDS-PAGE) and were then transferred onto a polyvinylidene fluoride (PVDF) membrane (Bio-Rad, Hercules, CA, USA). Following the blocking with 3% bovine serum albumin in PBS-T, the membrane was sequentially incubated with each of the primary

antibodies at 4°C for 16 h, and subsequently with horseradish peroxidase (HRP)-conjugated secondary antibody at 20°C for 1 h. Anti-E-cadherin polyclonal antibody (Abcam, Cambridge, MA, USA) and anti- Glyceraldehyde-3-Phosphate Dehydrogenase (GAPDH) polyclonal antibody (Santa Cruz Biotechnology) were used as primary antibodies. After repeatedly washing with PBS-T, the membrane was developed using Enhanced chemiluminescent (ECL) reagent (GE Healthcare, Uppsala, Sweden). The protein bands were visualized using Hyperfilm^{TM} ECL (GE Healthcare), and then quantitatively analyzed using Image J software (National Institute of Health).

Quantitative real-time PCR (qPCR)

Following cell harvest, the total RNA was extracted using the Ribospin^{TM} total RNA purification kit (GeneAll Biotechnology Co., Ltd., Seoul, Korea), according to the manufacturer's instructions. After cDNA was synthesized from the purified RNA using TOPScript^{TM} RT DryMIX (Enzynomics Co. Ltd., Daejeon, Korea) and dT 18 plus primer, quantitative real-time PCR was carried out for each gene using TOPreal^{TM} qPCR 2× PreMIX (SYBR Green with high ROX, Enzynomics Co. Ltd.) with specific primers on an Eco Real-Time PCR System (Illumina, San Diego, CA, USA). The primer sequences are summarized in Table S1. Each mRNA expression level was normalized to that of GAPDH, an endogenous control, and the relative fold-change in the EBs, compared to undifferentiated iPSCs, was determined using the $2^{-\Delta\Delta Ct}$ method.

Statistical Analysis

All assays were performed in triplicates, unless otherwise indicated. The data are shown as mean ± standard deviation. Statistical significance was calculated using Student's t-test.

References

Carpenedo, R.L., Sargent, C.Y., and McDevitt, T.C. (2007). Rotary suspension culture enhances the efficiency, yield, and homogeneity of embryoid body differentiation. Stem Cells *25*, 2224-2234.
